# A nontandem novel compound chimeric antigen receptor redirected to target CD20‐CD19 positive B‐cell acute leukemias and B‐cell lymphoma

**DOI:** 10.1002/ctm2.1743

**Published:** 2024-07-23

**Authors:** Vincent M DeStefano, Masayuki Wada, Kevin G Pinz, Rita Assi, Hongyu Zhang, Weijia Wang, Wenli Zhang, Darshi Shah, Yupo Ma, Huda Salman

**Affiliations:** ^1^ Research & Development Division iCell Gene Therapeutics Inc., Long Island High Technology Incubator Stony Brook New York USA; ^2^ Department of Medicine Indiana University School of Medicine Indianapolis Indiana USA; ^3^ Department of Hematology Peking University Shenzhen Hospital Shenzhen China; ^4^ Department of Advanced Diagnostic and Clinical Medicine Zhongshan People's Hospital Zhongshan People's Republic of China; ^5^ Brown Center for Immunotherapy Indiana University School of Medicine Indianapolis Indiana USA

Dear Editor,

A novel anti‐CD20, anti‐CD19 compound chimeric antigen receptor T‐cell therapy (cCAR) comprised of two distinct, fully functional CARs to independently target CD20 and CD19 surface antigens (SAs) in one application has been developed to potentially reduce relapsed/refractory (r/r) disease due to antigen escape. The CD20‐CD19 cCAR T‐cell construct has demonstrated remarkably precise cytotoxicity in 24 h co‐culture assays which resulted in >99% (2:1 and 5:1) ablation of REH cells, 79% (2:1) and 98% (5:1) ablation of K562‐CD19xp cells, 81% (2:1) and 87% (5:1) ablation of K562‐CD20xp cells, and >99% (2:1) ablation of primary patient B‐ALL‐25 cells (in vitro) as well as remarkable cytotoxicity (>99%) ablation of antigen‐positive target cells in NSG mice cohorts engrafted with REH, U937wt, and U937‐CD20xp cells treated with 1.0 × 10^7^ CD20‐CD19 cCAR T cells (in vivo).

CAR T cells have emerged as novel and effective treatments for B‐cell malignancies and non‐Hodgkin's lymphoma (NHL); however, both primary and secondary resistance have occurred,^1^ and relapse rates have been recorded in 65−85% of patients following CAR T treatment.^2^ Selective pressure has resulted in target antigen loss and may be overcome through targeting both the CD20 and CD19 antigens exclusively present on B cells.^3^ CD20 retains surface expression in >90% of B‐cell malignancies^4^ and CD19 is expressed in 80–100% of cases.^5^, ^6^, ^7^ Thus, our novel CD20‐CD19 cCAR T construct generated using a lentiviral vector, independently expresses each CAR unit in approximately equivalent ratios (Figure 1A). CAR T‐cell expression was evaluated with flow cytometry and analyzed using Kaluza software as previously described.^8^

**FIGURE 1:**
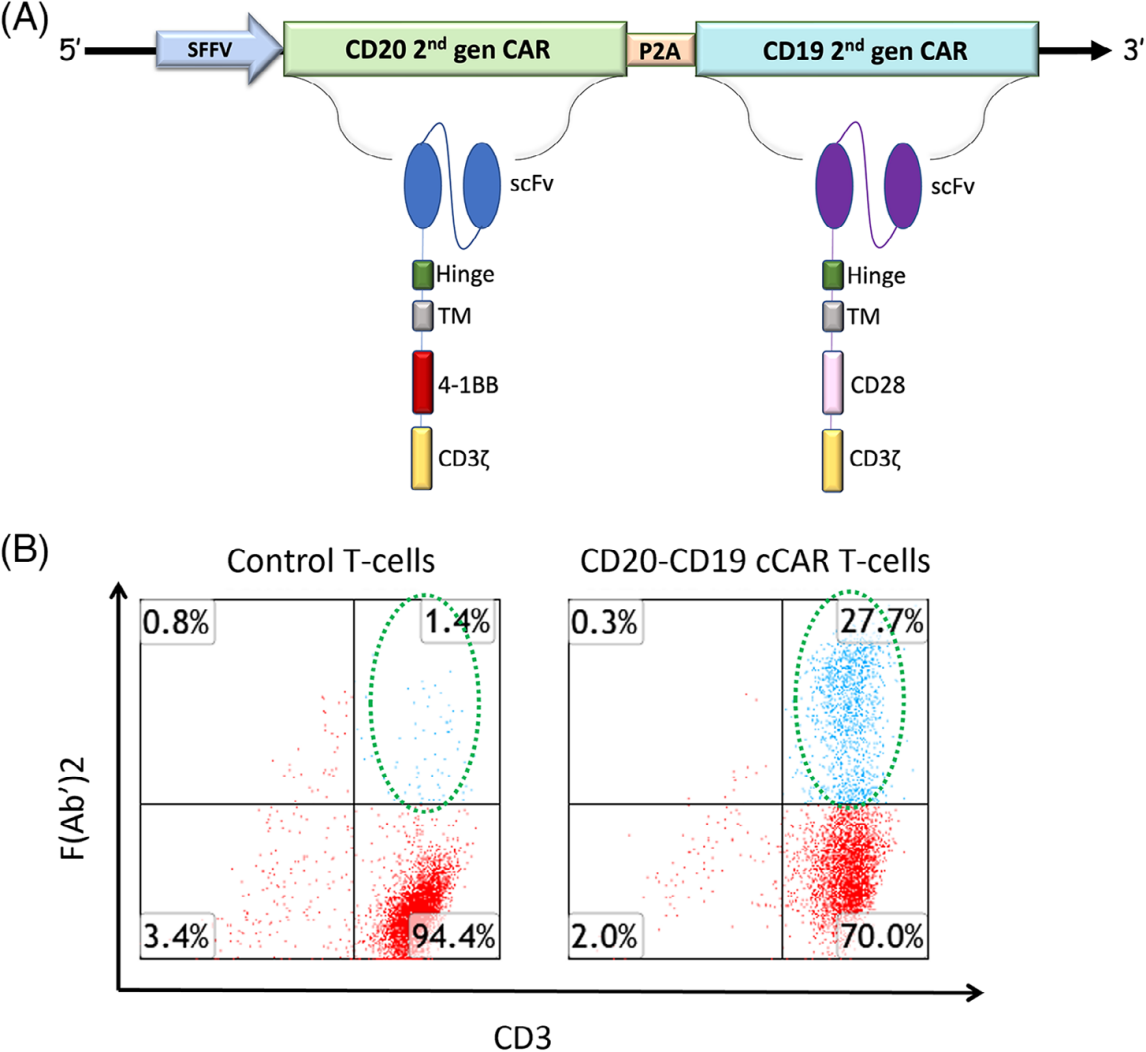
(A) Two discrete CAR units fused via a self‐cleaving P2A peptide: an anti‐CD20 CAR comprised of a CD8‐derived hinge, transmembrane (TM) region, 4‐1BB, and CD3ζ domain, as well as an anti‐CD19 CAR comprised of CD8‐derived hinge, TM region, CD28, and CD3ζ domain. A strong SFFV promoter was utilized to ensure the highly efficient expression of the CAR molecules. The CD28 domain uniquely induces rapid proliferation and persistence is further maintained by 4‐1BB. (B) Expression of CD20‐CD19 cCAR T cells. Buffy coat cells were activated for 3 days with anti‐CD3 antibody. Cells were transduced with either control vector (left panel), or CD20‐CD19 cCAR (right panel) lentiviral supernatant. After 3 days of incubation, cells were harvested and labelled for flow cytometry.

Human primary patient, antigen‐positive B‐ALL samples, as well as non‐disease peripheral blood mononuclear cells, were obtained using Stony Brook University's institutional review board‐approved protocol. Written informed consent was obtained from all donors. REH, K562, and U937 cell lines were obtained from ATCC and T cells were cultured as previously described.^8^ Two sets of NOD SCID gamma mice from the Jackson Laboratory were used, as previously described. Following irradiation and randomization, mice were injected with 1.0 × 10^6^ REH cells and treated with 1.0 × 10^7^ cCAR (*n *= 5) or control (*n *= 5) T cells 24 h after engraftment. In the second set, mice were injected with 1.0 × 10^6^ U937‐20xp (*n *= 4) or U937wt (*n *= 4) cells and treated with 1.0 × 10^7^ cCAR T cells 24 h later. Images were obtained and analyzed as previously described.^8^

CD20‐CD19 cCAR T cells were generated and CAR expression was recorded at approximately 27.7% by Day 3 (D3) (Figure 1B). Co‐culture killing assays targeting REH (CD19^+^ CD20^+^), K562‐CD20xp (CD19^−^ CD20^+^), K562‐CD19xp (CD19^+^ CD20^−^), and primary patient B‐ALL samples (CD19^+^ CD20^+^), were performed. In 24 h experiments, the cCAR T therapy demonstrated profound lysis of REH cells (>99%) at effector to target (E:T) ratios of 2:1 and 5:1 (Figure 2A). Targeting synthetic K562‐CD20xp cells resulted in robust anti‐tumour lysis with approximately 81% and 87% depletion in 2:1 and 5:1 24 h co‐cultures, respectively (Figure 2B). Killing assays of K562‐CD19xp cells demonstrated profound anti‐tumour lysis with approximately 79% and 98% depletion in 2:1 and 5:1 24 h co‐cultures, respectively (Figure 2C). Primary patient B‐ALL cell samples (CD19^+^ CD20^+^) were incubated with P2A control or cCAR T cells which showcased total target ablation (>99%) at the E:T ratio of 2:1. Antigen negative controls were not lysed when cultured with our therapy demonstrating target specificity and no off‐target effects (Figure 2D).

**FIGURE 2:**
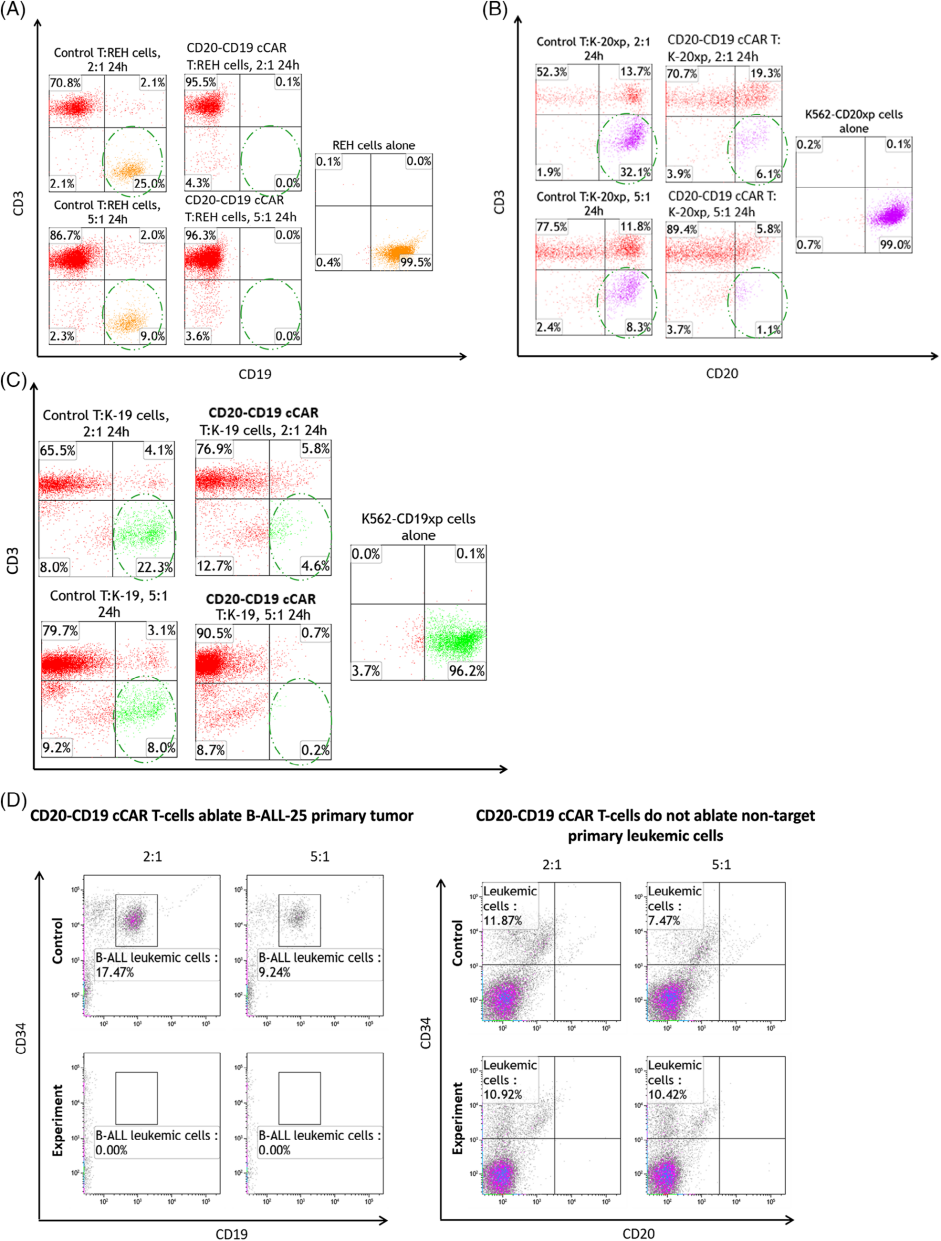
(A) CD20‐CD19 cCAR T cells completely lyse REH cells in vitro. Co‐culture experiments were performed at E:T ratios of 2:1 or 5:1 for 24 h and analyzed by flow cytometry for CD19 and CD3. Each assay consists of REH target cells alone (right panel), control T cells (left panels) and CD20‐CD19 cCAR T cells (centre panels). Target cells are represented as orange dots. (B) CD20‐CD19 cCAR completely lyse K562‐20xp cells in vitro. Co‐culture experiments were performed at E:T ratios of 2:1 or 5:1 for 24 h and analyzed via flow cytometry for CD20 and CD3. Each assay consists of K562‐20xp target cells alone (right panel), control T cells (left panels) and CD20‐CD19 cCAR T cells (centre panels). Target cells are represented as magenta dots. (C) CD20‐CD19 cCAR T completely lysed K562‐19xp cells in vitro. Co‐culture experiments were performed at E:T ratios of 2:1 or 5:1 for 24 h and were directly analyzed by flow cytometry for CD19 and CD3. Each assay consists of K562‐19xp target cells alone (right panel), control T cells (left panels) and CD20‐CD19 cCAR T cells (centre panels). Target cells are represented as light green dots. (D) CD20‐CD19 cCAR T cells ablate primary B‐ALL cells and verify no off‐target leukemic cells. Co‐cultures against primary B‐ALL (CD19^+^ and CD20^+^) leukemic blasts (B‐ALL‐25) and primary leukemic cells (CD19^−^,CD20^−^, and CD34^+^). FACS analysis of co‐cultures against B‐ALL‐25 (left panels) and negative control primary cell co‐culture (right panels).

CD20‐CD19 cCAR T cells exhibited profound anti‐tumour activity in vivo which was evaluated by: (1) IVIS‐fluorescent imaging, (2) promulgation of superior survival outcomes, and (3) FACS analysis of peripheral blood cells. NSG mice were sub‐lethally irradiated and engrafted with 1.0×10^6^ luciferase expressing REH cells and treated with 1.0 × 10^7^ CD20‐CD19 cCAR T cells or vector control T cells, 6 days post‐REH cell injection. On days 5, 9, 12, and 16, mice were injected with RediJect D‐Luciferin and subjected to IVIS imaging. Treatment achieved 98% lysis of tumour cells by D12 and >99% ablation by D16 and fluorescent imaging indicated significantly improved survival outcomes in mice treated with cCAR (Figure 3A, B). The remarkable depletion of target cells by cCAR was confirmed via FACS analysis of peripheral blood samples of both experimental and control mice (Figure 4A, B).

**FIGURE 3:**
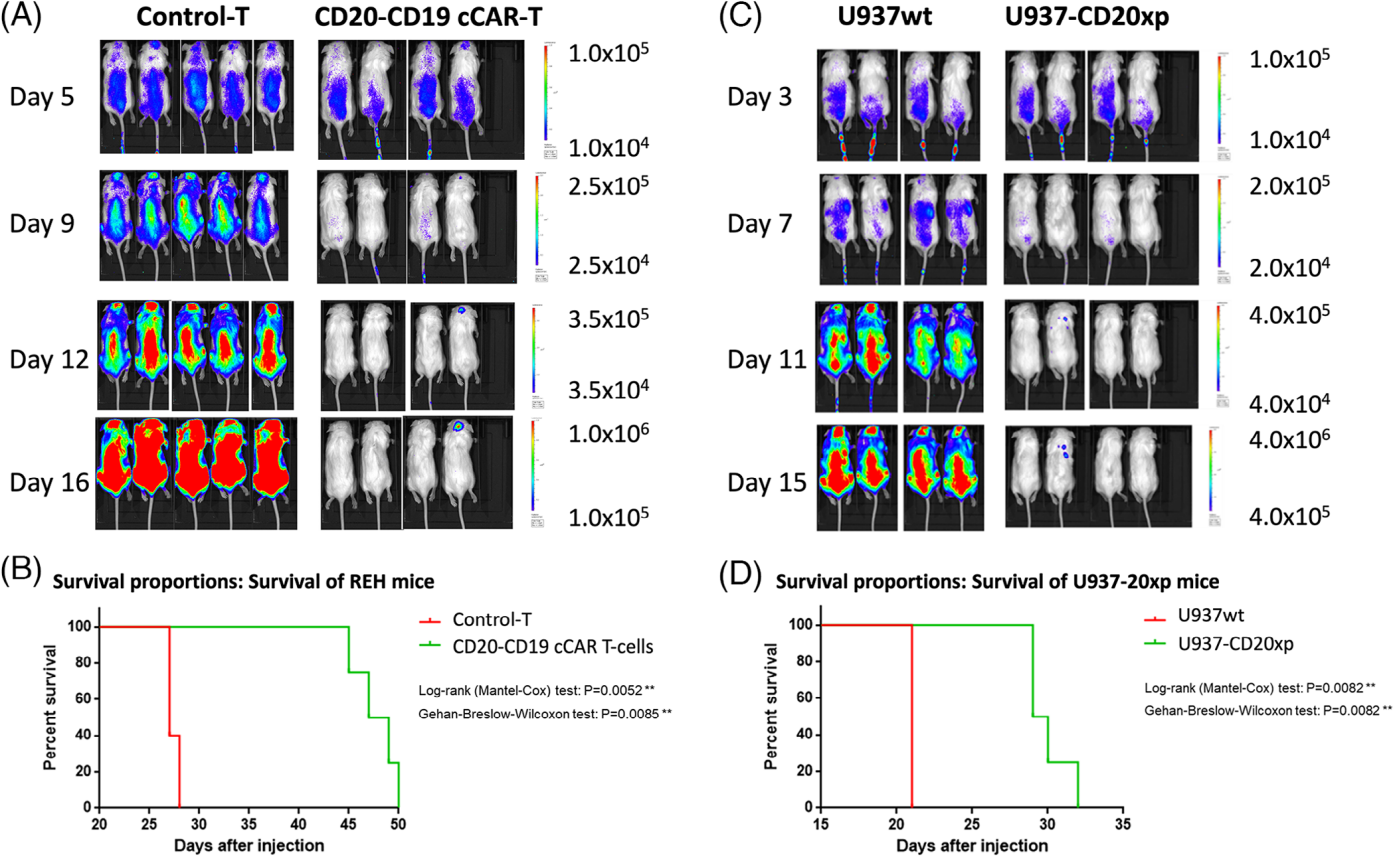
(A) CD20‐CD19 cCAR T cells demonstrate cytotoxic effects in vivo against the REH tumour cell line (CD20^+^ and CD19^+^). Dorsal view. (B) CD20‐CD19 cCAR T cells significantly prolong survival in treated mice. Survival data of mice injected with REH tumour cells, and either control (red) or cCAR (green) T cells. Mice were monitored daily and sacrificed upon paralysis. CD20‐CD19 cCAR T‐treated mice demonstrated significantly improved survival compared to controls (log‐rank Mantel‐Cox, *p* = 0.0052; Gehan–Breslow–Wilcoxon tests *p* = 0.0085). (C) CD20‐CD19 cCAR T cells demonstrated profound cytotoxic effects in vivo against U937‐20xp cells; no killing of U937wt cells. Dorsal view. (D) CD20‐CD19 cCAR T cells promulgate superior survival outcomes of U937‐CD20xp engrafted mice. Survival data of mice injected with U937wt tumour cells (control; red) or CD20‐CD19 cCAR T (control; green). Mice were monitored daily and sacrificed upon paralysis. Significantly increased survival was demonstrated in the experimental group (log‐rank Mantel‐Cox, *p* = 0.0082; Gehan–Breslow–Wilcoxon test *p* = 0.0082).

**FIGURE 4:**
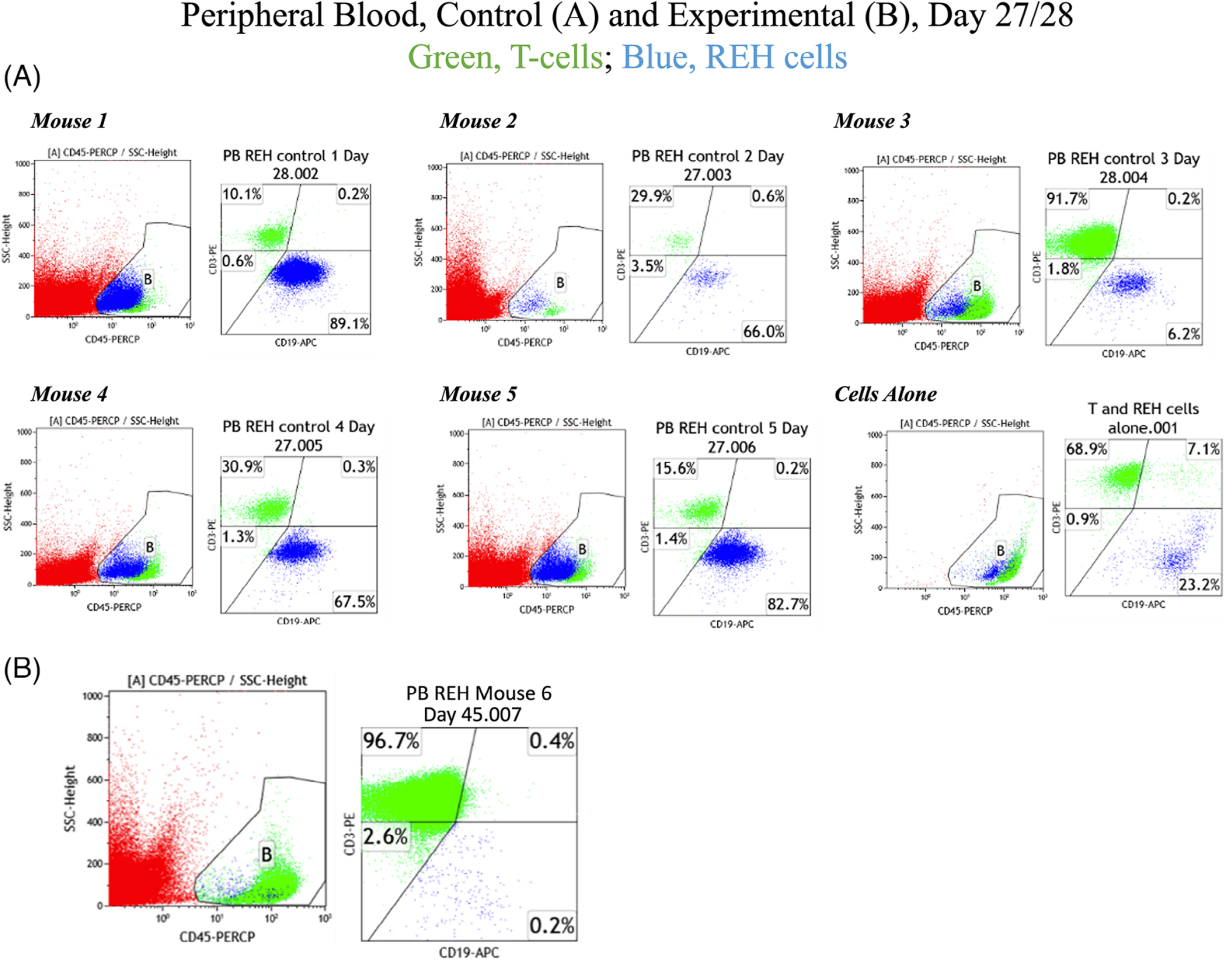
(A) FACS analysis of tumour persistence and expansion in the control mice group. Mice were sacrificed upon paralysis. Peripheral blood, collected at sacrifice, labelled with CD3‐PE, CD45‐PerCp, CD19‐APC. CD45^+^ selection (left panel); CD3 (T cells) vs. CD19^+^CD20^+^ (REH cells), CD45^+^ cells (Gate B) only (right panel). Evidence of significant tumour burden (blue dots) can be observed in all control mice. (B) FACS analysis of tumour persistence and expansion in a CD20‐CD19 cCAR T‐treated mouse (PB REH mouse 6). The experimental mouse was euthanized, and the peripheral blood was analyzed by FACS. Peripheral blood, collected at sacrifice, labelled with CD3‐PE, CD45‐PerCp, CD19‐APC. CD45^+^ selection (left panel); CD3 (T cells) vs. CD19^+^CD20^+^ (REH cells), CD45^+^ cells (Gate B) only (right panel). Remarkable depletion of tumour cells (blue dots) was observed for this CD20‐CD19 cCAR T‐treated mouse.

Specificity and cytotoxicity were further confirmed via targeting of U937 wild‐type (U937wt) and U937 cells stably expressing CD20 (U937‐CD20xp). NSG mice were sub‐lethally irradiated and intravenously injected with 1.0 × 10^6^ luciferase‐expressing U937wt (*n = 4*) or U937‐CD20xp (*n = 4*) cells (D0) and treated with 1.0 × 10^7^ cCAR T cells 4 days post‐tumour engraftment. On days 3, 7, 11, and 15, mice were injected with RediJect D‐Luciferin and subjected to IVIS imaging. CD20‐CD19 cCAR T cells displayed discrete CD20 antigen targeting in vivo. A significant difference in tumour burden was recorded by D7 (*p* < 0.01) and further evident by D15 with >99% depletion due to cCAR treatment (Figure 3C). Significant survival improvement was demonstrated (Figure 3D).

In this preclinical study, the novel CD20‐CD19 cCAR T construct demonstrated remarkable efficacy and target antigen specificity both in vitro and in vivo. As such, our CD20‐CD19 cCAR T cells may present an enhanced therapeutic approach via dual targeting of CD19^+^ and/or CD20^+^ antigens in B‐cell malignancies and NHL, where 30−60% of patients relapse mainly due to antigen escape or downregulation.^9^ Cancer cells may lose SAs following natural or therapy‐induced selective pressure and these antigen‐loss variants are often the cause of therapy‐resistant relapse. Relapse can also occur from a decrease in SA density and lineage switch. CD19 and CD20 antigen loss in acute lymphoblastic leukaemia and chronic lymphocytic leukaemia, respectively, are well‐documented in this regard. In particular, exon 2 of CD19 was frequently spliced out, leading to the disappearance of the CD19 epitope.^10^

Patients with CD19^−^ relapses have poor prognoses and novel approaches, such as our cCAR are direly needed. This data demonstrates the feasibility of CD20‐CD19 cCAR T to treat CD20^+^ or CD19^+^ malignancies. A primary mediastinal diffuse large B‐cell lymphoma patient was compassionately dosed with CD20‐CD19 cCAR T and achieved CR; data to be presented at the conclusion of the trial.

## AUTHOR CONTRIBUTIONS

Vincent M DeStefano: Conceptualization, methodology, writing—original draft, writing—review & editing, visualization, supervision. Masayuki Wada: Conceptualization, methodology, data curation, writing—original draft, visualization. Kevin G Pinz: Conceptualization, methodology, data curation, writing—original draft, visualization. Rita Assi: Writing—original draft. Hongyu Zhang: Data curation, writing—original draft. Weijia Wang: Data curation, writing—original draft. Wenli Zhang: Data curation. Darshi Shah: Writing—original draft. Yupo Ma: Conceptualization, methodology, writing—original draft, writing—review & editing, supervision. Huda Salman: Conceptualization, writing—original draft, writing—review & editing, supervision.

## CONFLICT OF INTEREST STATEMENT

KGP, MW, and YM are co‐inventors of this technology and hold the patents relevant to the contents of this manuscript. YM is a founder and Chairman of iCell Gene Therapeutics, Inc. MW, KGP, VMD, DS, and YM are employees of iCell Gene Therapeutics Inc.

## FUNDING INFORMATION

No funding to report.

## ETHICS STATEMENT

The study was approved through Stony Brook University's institutional review board (IRB).

## Data Availability

The data that support the findings of this study are available on request from the corresponding author.
